# Lubricating Polymer Gels/Coatings: Syntheses and Measurement Strategies

**DOI:** 10.3390/gels10060407

**Published:** 2024-06-19

**Authors:** Panpan Zhao, Jacob Klein

**Affiliations:** Department of Molecular Chemistry and Materials Sciences, Weizmann Institute of Science, Rehovot 76100, Israel

**Keywords:** polymer gels/coatings, lubrication mechanism, synthesis strategy, friction measurements

## Abstract

Straightforward design and long-term functionality for tribological considerations has prompted an extensive substitution of polymers for metals across various applications, from industrial machinery to medical devices. Lubrication of and by polymer gels/coatings, essential for ensuring the cost-effective operation and reliability of applications, has gained strong momentum by benefiting from the structural characteristics of natural lubrication systems (such as articular cartilage). The optimal synthetic strategy for lubricating polymer gels/coatings would be a holistic approach, wherein the lubrication mechanism in relation to the structural properties offers a pathway to design tailor-made materials. This review considers recent synthesis strategies for creating lubricating polymer gels/coatings from the molecular level (including polymer brushes, loops, microgels, and hydrogels), and assessing their frictional properties, as well as considering the underlying mechanism of their lubrication.

## 1. Introduction

Nature presents numerous examples of systems which show superlubrication, energy conservation, and elegance in their design, particularly articular cartilage (friction coefficient μ ≈ 0.001 or less under physiological pressures up to 10 MPa or more) [1,2]. Learning from nature, a diverse range of lubricating materials have been developed, encompassing nanoparticles [3,4,5], micro-/nanogels [6,7], ionic liquids [8], hydrogels [9], and surfactants [10]. Polymers, which serve as building blocks to fabricate materials with a lower density, enhanced mechanical properties, and non-toxic nature, have critical applications in a wide variety of fields ranging from biomedical devices to tissue engineering, drug delivery, and soft robotics [11,12,13]. Thus far, various types of polymers with hydrophilic, hydrophobic, or amphiphilic properties have been explored, with microstructures including polymer loops, brushes, micelles, and hydrogels [14,15,16]. Despite extensive progress, challenges remain of achieving good lubricity, a vital characteristic favorable to the reliable functioning of engineering and medical applications.

Lubrication, the process that reduces the friction force between two opposing surfaces sliding on each other, is a key function to ensure the proper operation of diverse biomedical devices such as implants and sensors, as well as medical treatments as in osteoarthritis (OA) [17,18,19]. There are three different types of lubrication modes in the Stribeck curve put forward by Stribeck [20] and Hersey [21] long ago, namely boundary, hydrodynamic, and mixed lubrication. Under low velocity and high load conditions, so that a thin or unstable tribofilm whose thickness is less than the surface roughness is formed, and the sliding surface asperities collide, is defined as boundary lubrication [22,23]. Over the past decades, the friction between polymeric surfaces in aqueous environments has attracted special interest. Numerous studies have elucidated a lubrication mode in aqueous systems, defined as the hydration lubrication mechanism, whereby sub-nanometer-thick hydration layers are capable of withstanding substantial normal stresses without being squeezed out, meanwhile retaining persistent fluidity under shear [24,25]. This mechanism can thus significantly diminish the friction between sliding surfaces bearing hydrated polymers [26,27]. Many studies have focused on preparing lubricating polymer gels/coatings based on the hydration lubrication mechanism; however, their detailed lubrication mechanism over several length scales is still not fully clear.

The surface topology, referring to the geometric characteristics of a surface, is anticipated to modulate lubrication between solid surfaces. Tuning the surface topology of polymer gels/coatings at the molecular level may lead to a deeper understanding of their lubrication and in turn facilitate the customization of materials for lubricated applications. In this review, we discuss four major synthetic strategies for modulating the surface topology of polymer gels/coatings at the molecular level for lubrication properties: free radical polymerization (FRP), controlled/living radical polymerization (CRP), click chemistry, and supramolecular assembly, together with the corresponding measurements characterizing their frictional behavior. Finally, we highlight future directions and challenges for the facile and universal synthetic strategies of lubricating polymer gels/coatings.

## 2. Measurement Strategies for Lubrication

### 2.1. Surface Force Balance (SFB)

**Figure 1:**
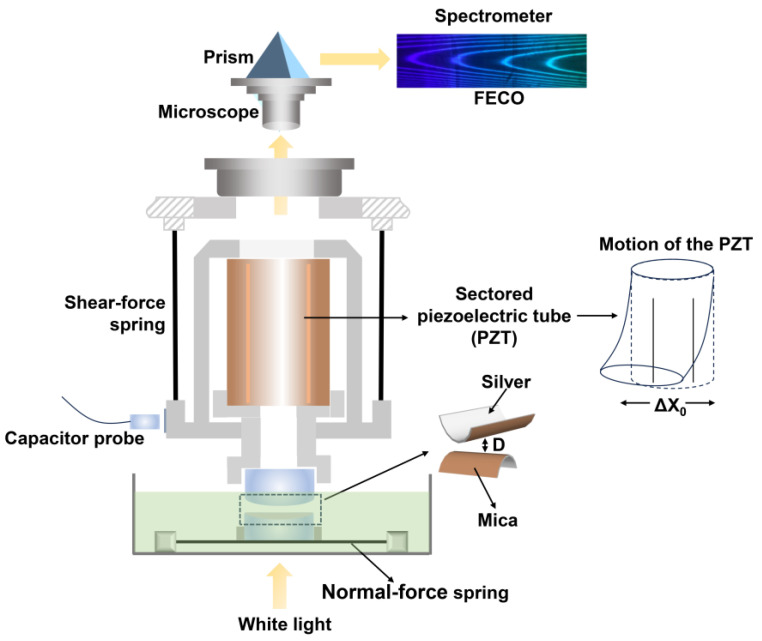
A detailed schematic of the surface force balance (SFB) employed for directly detecting the normal and shear forces between mica surfaces [29]. Reproduced with permission from Ref. [29]; Rights managed by AIP Publishing.

A surface force balance (SFB) is a device used for directly measuring both normal (F_n_) and shear (F_s_) forces exerted by or on surfaces as a function of their absolute separation D, enabling a coefficient of friction COF (µ) to be examined according to Amontons’ law F_s_ = μF_n_. Figure 1 shows the schematic of the SFB developed by the Klein group [28] to illustrate the general principle [29]. The wavelengths of fringes of equal chromatic order (FECO) evolving from multiple beam interferometry determines the D between half-silvered mica sheets within a 1–2 Å resolution, as well as the true contact area between the surfaces. Surface deformations occurring in the contact area can also be monitored by visualized fringes. A sectored piezoelectric tube (PZT) mounted onto shear-force springs drives the lateral motion of the upper surface by controlling the expansion/contraction of sectors through applied voltages. The shear forces F_s_ (with a resolution of ca. 100 nN) are calculated using Hooke’s law via recording the bending of shear-force springs, and this bending is tested by an air-gap capacitance probe. The normal forces F_n_ (with a resolution of ca. 10 nN) are measured via the bending of a normal-force spring determined by monitoring the wavelength variation in the FECO.

### 2.2. Atomic Force Microscopy (AFM)

**Figure 2:**
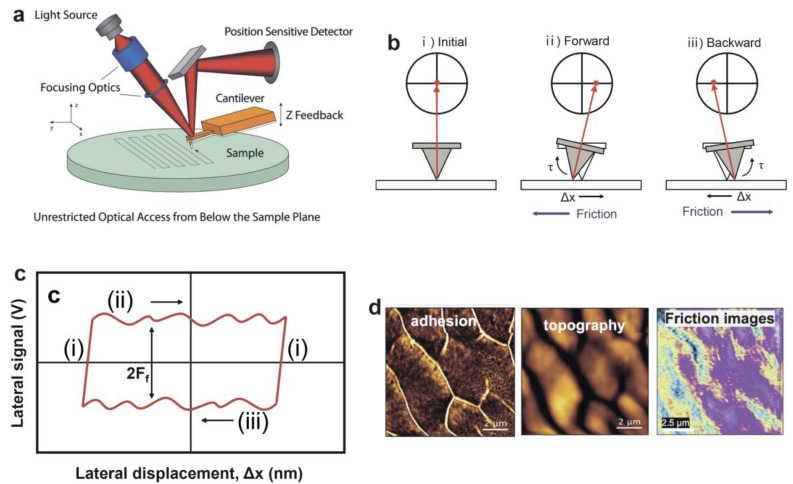
(**a**) Sketches of an AFM. The cantilever twisting is monitored by a photodetector, which converts the laser point movement into surface topography [34]. (**b**) The operating principle of the lateral force microscope (LFM). The probing direction is orthogonal to the cantilever axis to allow the cantilever to twist during sliding. (**c**) The schematic diagram of a typical friction loop. The Y axis corresponds to the lateral signal on the photodetector as the tip scans on the sample surface [30]. (**d**) QI images of adhesion, height, and friction maps of AgP(Am-co-AAc) DN hydrogel in water [32]. (**a**) Reproduced with permission from Ref. [34]; Copyright © 2009 Elsevier Ltd. (**b**,**c**) Reproduced with permission from Ref. [30]; Copyright © 2010 Elsevier Ltd. All rights reserved. (**d**) Reproduced with permission from Ref. [32]; Copyright © 2023 The authors.

Atomic force microscopy (AFM) provides valuable insights into the surface morphology of target materials at the nano-scale, through which height and lateral deflection images are taken simultaneously. In particular, a lateral force microscope (LFM), whose principle is similar to that of contact-mode AFM, can be used for analyzing frictional properties quantitatively [30,31]. When the tip scans across the sample surface orthogonal to the cantilever axis under a given normal force, the consequent lateral deflection of the cantilever is additionally monitored by the optical deflection technique to determine the distribution of the lateral force across the sample surface by knowledge of the torsional spring constant (Figure 2a,b). Figure 2c shows a friction loop: (i) the tip remains stationary on the surface; (ii) the probe moves to the right, and the lateral deviation of the cantilever is measured by the photodetector; (iii) the probe goes to the left and the signal is recorded by the photodetector. The sliding friction between sample and tip is represented by the half width of the friction loop in the vertical direction. Using a UV-triggered FRP approach, Lee et al. [32] prepared an agarose/poly(acrylamide-co-acrylic acid) (AgP(Am-co-AAc)) double network (DN) hydrogel. The height, adhesion, and frictional images of the DN hydrogel were obtained from LFM measurement, as shown in Figure 2d. Sader’s method [33] was employed to assess the normal spring constant, meanwhile a noncontact thermal noise method was applied for lateral calibration. Friction images were then derived from lateral force images acquired in contact mode.

### 2.3. Tribometer

**Figure 3:**
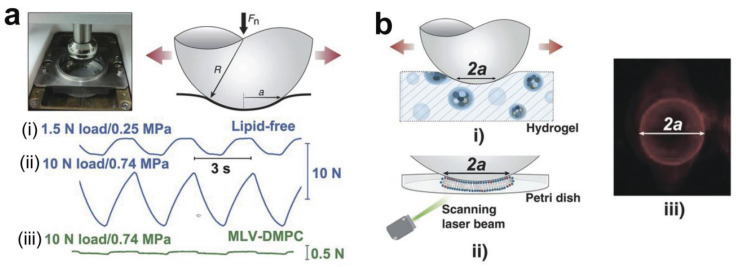
(**a**) The schematic diagram of a UMT tribometer, and corresponding sliding configuration and typical friction-versus-time traces. (**b**) The method of confirming the contact area (radius *a*) with the sliding steel sphere monitored by a photomultiplier tube [35]. Reproduced with permission from Ref. [35]; Copyright © 2020, The American Association for the Advancement of Science.

A tribometer is a device used to measure the friction between surfaces in contact, particularly in tribology, which is the study of friction, wear, and lubrication, including pin-on-disc, ball-on-disc, and reciprocating tribometers. It is an essential tool in simulating and quantifying the interactions between surfaces in contact under various conditions, providing valuable insights into their frictional behavior and wear resistance. As an example, a ball-on-disk tribometer equipped with normal and friction force sensors, as well as a capacitance-based sensor, was designed to ascertain the frictional force, wear, and indentation depth of materials under an applied load (Figure 3a). The contact radius *a* could be calculated via Hertzian contact mechanics or directly measured (Figure 3b). With the contact radius determined, the contact area was calculated using the formula A = Π*a*^2^. The sliding friction F_s_ can be obtained from its value in the plateau region of the friction traces across a range of loads F_n_ (1.5–50 N, corresponding to mean contact pressures in the range of 0.25–1.53 MPa), allowing for the calculation of the COF (μ) as the ratio of F_s_ to F_n_.

Notably, SFB possesses certain advantages in directly measuring the absolute distance D with an Å-level resolution between surfaces and detecting both shear and normal forces with a higher resolution than AFM. This capability allows researchers to interrogate the lubrication mechanisms and properties of materials at a sub-nanometer scale that are difficult to access through other methods such as a tribometer. However, SFB is a challenging technology that faces obstacles such as surface contamination, solvent evaporation, and thermal fluctuations, which can affect the reliability and reproducibility of the results.

## 3. Chemical Processes Utilized to Synthesize Lubricating Polymeric Materials

The main approaches for the synthesis of lubricating polymer gels/coatings are free radical polymerization (FRP), controlled/living radical polymerization (CRP), click chemistry, and supramolecular chemistry. To elucidate lubrication properties, various force measurement techniques such as atomic force microscopy (AFM), tribometer, and surface force balance (SFB) (or equivalently surface force apparatus (SFA)) have been extensively employed [27,28,29]. Table 1 summarizes the synthesis methods and friction measurements with different polymer-based lubricants.

**Table 1:** Summary of synthesis methods and friction measurements with different polymer-based lubricants.

Polymer-Based Lubricants	Synthesis Methods	Measurements	µ	Ref.
Homo-oligomeric phosphocholinated micelles (OMDPC)	FRP	SFB	0.002 ± 0.001	[36]
PC lipids/pHEMA hydrogels	FRP	Tribometer	≈0.01	[35]
Lubricin mimicking ABA bottle-brush polymer	ATRP	SFB	0.0025 in pure water and 0.0115 in PBS	[37]
Agarose/poly(acrylamide-co-acrylic acid)	FRP	AFM	~0.01 to 0.02	[32]
Bottle-brush polymer (BPHEMA)	RAFT	Tribometer	~0.3	[38]
P(EO-co-AGE)-b-PEO-b-P(EO-co-AGE) triblock copolymers	Click chemistry	SFB	0.002 ± 0.001	[39]
Silk fibroin/PAAm/PVA hydrogel	Supramolecular assembly	Tribometer	~0.08	[40]
β-CD-PMPC polymer	Supramolecular assembly	Tribometer	0.024–0.028	[41]

### 3.1. Free Radical Polymerization (FRP)

**Figure 4:**
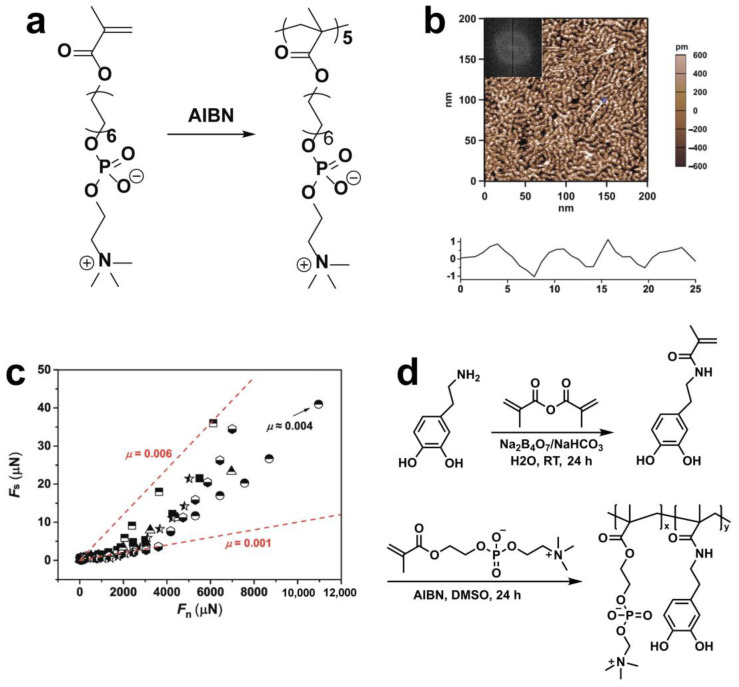
(**a**) Synthesis procedure of oligo(1,2-methacryloyldodecyl phosphorylcholine) (OMDPC) micelles via free radical polymerization (FRP) [36]. (**b**) AFM images of OMDPC micelles-coated mica surfaces [36]. (**c**) Frictional force (F_s_) versus normal force (F_n_) between two mica surfaces across an OMDPC micelles solution measured by SFB [36]. (**d**) Synthesis routes of the DMA monomer and poly(MPC-*co*-DMA) copolymers [43]. (**a**–**c**) Reproduced with permission from Ref. [36]; Copyright © 2020 American Chemical Society. (**d**) Reproduced with permission from Ref. [43]; Copyright © 2024, The authors.

Throughout free radical polymerization (FRP), free radicals are generated through the decomposition of initiators, including thermal initiators (e.g., hydroperoxides, persulfates) or photoinitiators (e.g., benzophenone, thioxanthone/amine), which then initiate the polymerization process by attacking the double bonds of monomers [42]. Homo-oligomeric phosphocholinated micelles (OMDPCs) were prepared by Lin et al. through FRP using 1,2-(methacryloyloxy)dodecyl phosphorylcholine (MDPC) as a monomer, azobis(isobutyronitrile) (AIBN) as the thermal initiator (Figure 4a) [36]. The atomic force microscope (AFM) micrograph of the OMDPC homo-oligomer (Figure 4b) exhibited wormlike micelles with the center-to-center distance of about 5.0 nm on the mica substrate. A surface force balance (SFB) was applied to monitor the normal and shear forces between mica surfaces coated with OMDPC micelles as a function of their absolute separation distance (D) with an angstrom-level resolution. The shear force (F_s_) vs. normal force (F_n_) of OMDPC micelles-coated mica surfaces is summarized in Figure 4c. The OMDPC micelles exhibited a low friction coefficient (µ = 0.002 ± 0.001) up to the normal stress (P) of about 3 MPa in this study. This superlubrication observed here can be ascribed to hydration lubrication facilitated by the abundant hydration of the zwitterionic phosphocholine groups positioned at the surfaces of the dense-packed wormlike OMDPC oligomer micelles. 

In a different realization using the FRP approach, a catechol-functionalized polyzwitterion comprised of hydrophilic 2-methacryloyloxyethyl phosphorylcholine (MPC) and reactive oxygen species (ROS)-scavenging dopamine methacrylamide (DMA) monomers, termed p(MPC-co-DMA), was synthesized for treating ocular dryness [43]. The synthesis process involved straightforward thermal-triggered FRP, utilizing AIBN as an initiator and DMSO as a solvent, as depicted in Figure 4d. PMPC with a high hydrophilicity can serve as an osmoprotectant from tear desiccation; however, it shows limitations in interfacial retention due to its neutral charge. The DMA residues in p(MPC-co-DMA) can provide a robust mucoadhesion on the ocular surface via Michael addition and/or Schiff base reaction, thus extending the ocular retention time. The coefficient of friction (COF) was determined using a universal testing machine equipped with a coefficient of friction fixture, and was calculated based on the forces required to initiate motion (static friction) and maintain motion (kinetic friction). The COF of p(MPC-co-DMA)-coated silicon tube (mimicking the ocular surface) was found to be 0.37, lower than that of a pMPC-deposited surface (COF = 0.66) after three cycles, which is due to the enhanced stability of the coating against shear stress by the high adhesive strength of the catechol residues in DMA.

**Figure 5:**
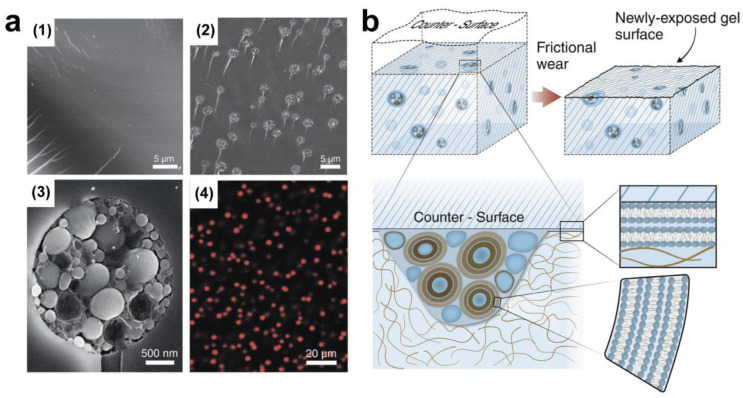
(**a**) SEM images of freeze fracture surfaces of lipid-free (**1**) and DMPC vesicles-incorporating (**2**) pHEMA hydrogels, the magnifying image of single microreservoir (**3**) from DMPC vesicles-incorporating pHEMA hydrogels, and (**4**) the confocal microscopy image of the fluorescence labeled lipid-incorporating hydrogel, illustrating the distribution of the lipid microreservoir. (**b**) The self-lubricating mechanism diagram of lipid-incorporating hydrogels. As friction wears away the hydrogel surface containing lipid vesicles in microreservoirs, new microreservoirs of lipids become exposed. This facilitates the formation of lipid boundary layers on the surfaces, reducing friction through the strong hydration of the lipid headgroups. Reproduced with permission from Ref. [35]; Copyright © 2020, The American Association for the Advancement of Science.

An additional application of this strategy concerns lubrication by hydrogels. Generally, hydrogels with 3D crosslinked hydrophilic polymer networks contain abundant water, and thus are slippery through hydrodynamic lubrication [9]. Poly(acrylamide) (PAAm) [44,45], poly(MPC-co-SBMA) copolymer (MPC: 2-methacryloyloxyethyl phosphorylcholine; SBMA: 2-(Methacryloyloxy)ethyl]dimethyl-(3-sulfopropyl) (sulfobetaine methacrylate)) [46], and poly(acrylic acid-co-acrylamide) (P(AA-co-AAm)) [47,48] hydrogels have been developed as lubrication materials via the copolymerization of monomers and crosslinkers using KPS (potassium persulfate) or APS (ammonium peroxodisulfate) as radical initiators. Lin et al. [35] synthesized different pHEMA hydrogels incorporated with PC lipids by the copolymerization of 2-hydroxyethyl methacrylate (HEMA) monomers and ethylene glycol dimethacrylate (EGDMA) crosslinkers. The distribution of PC lipid microreservoirs in the pHEMA hydrogels is depicted in Figure 5a. Friction measurements were conducted using a ball-on-disk tribometer, as shown in Figure 3a. The remarkable (up to 99%) decrease in friction observed in PC lipids/pHEMA hydrogels, relative to lipid-free gels, is ascribed to a lipid-based boundary layer exposed at the surface of the hydrogel. This acts via hydration lubrication at the exposed lipid headgroups at the slip plane, which undergoes continuous reconstruction as the hydrogel wears, facilitated by the gradual release of lipids from micro-reservoirs in the bulk gel (Figure 5b).

Photoinduced FRP is a process where the polymerization of vinyl monomers to form polymers is initiated by exposure to light. This method is commonly used in the manufacturing of various materials, including coatings, adhesives, and plastics. By using light as the trigger for polymerization, this method offers advantages such as precise control over the reaction, the ability to initiate polymerization in specific regions or patterns, low energy consumption, and wide adaptability over thermal polymerization [49,50]. Through this method, a vertical and horizontal dual gradient hydrogel-filled anisotropic tubular polymer skeleton (COF ≈ 0.0036) [51], hydrogel–elastomer hybrid surface [52], and GelMA-poly (N-Hydroxyethyl acrylamide) (GelMAPHEAA) chemical and physical network [53] were constructed.

**Figure 6:**
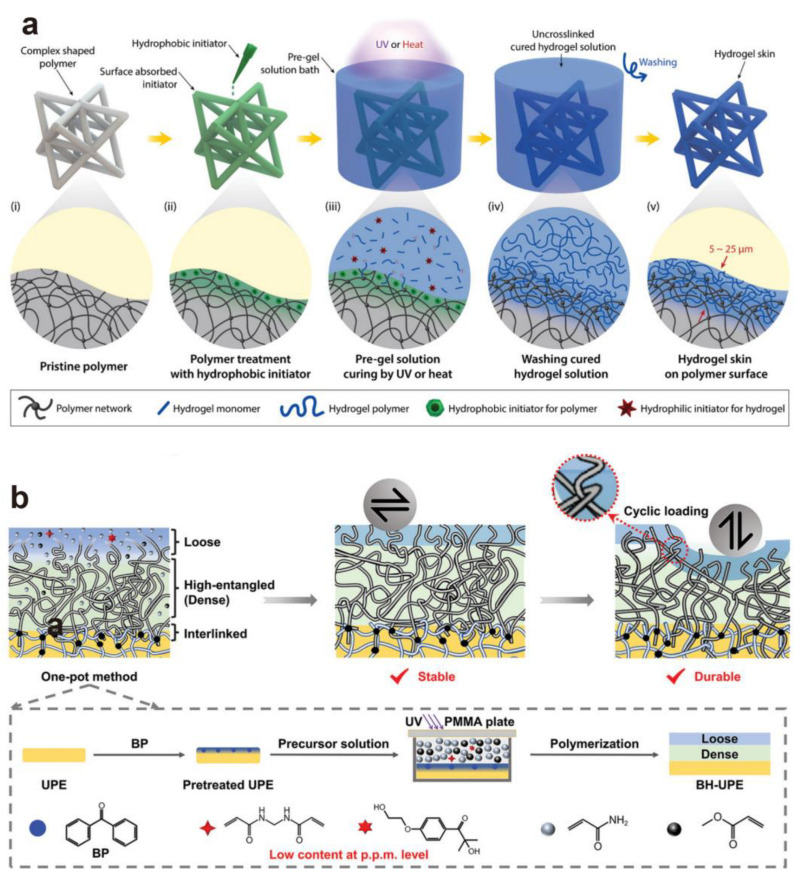
(**a**) The hydrogel “skin” preparation process is outlined schematically as follows: initially, polymer scaffolds undergo treatment with a hydrophobic initiator organic solution; subsequently, a hydrogel precursor solution with hydrophilic initiators was deposited onto the surface of these pretreated polymer scaffolds; following the curing of monomers and removing unreacted materials, thin-skin hydrogels are finally constructed on the surface of the scaffold through the interpenetrating interaction between hydrogel and polymer scaffolds [11]. (**b**) The facile three-in-one strategy of constructing an interlinked hydrogel–UPE interface [54]. (**a**) Reproduced with permission from Ref. [11]; © 2018 Wiley-VCH Verlag GmbH & Co. KGaA, Weinheim. (**b**) Reproduced with permission from Ref. [44]; © 2024 Wiley-VCH GmbH.

Innovatively, a simple yet effective method to incorporate hydrophilic polymers into the surface of virous polymers (PU, PVC, and nitrile rubber, etc.) with arbitrary shapes forming naturally integrated “hydrogel skins” was reported (Figure 6a) [11]. It based on a combination of surface-absorbed hydrophobic initiators for the polymer substrates and hydrophilic initiators for the hydrogel precursor solutions. The process involves initially treating pristine polymer scaffolds with hydrophobic photo- or thermo-initiators, such as benzophenone, 4-methyl benzophenone, or benzoyl peroxide, in organic solvents to facilitate absorption. Subsequently, these treated scaffolds are fully submersed in a hydrogel precursor solution containing hydrophilic initiators and monomers in an aqueous medium. Upon triggering polymerization of the precursors via UV or heat, the absorbed hydrophobic initiators act as grafting agents, facilitating the physical crosslinking of hydrogel polymers with substrate polymer chains and serving as oxygen scavengers to mitigate the oxygen inhibition effect. Concurrently, the hydrophilic initiators catalyze monomer polymerization, resulting in hydrogel formation within and above the surface-bound diffusion layer of the polymer scaffolds. Importantly, the insolubility of hydrophobic initiators in water inhibits their diffusion towards the aqueous hydrogel precursor solution, thereby constraining reactions (e.g., polymerization, interpenetration, and grafting) within the surface-bound diffusion layer. This union of selective and bounded diffusion of hydrophobic initiators fosters the creation of hydrogel skins through an interfacial interpenetration process.

As an example, an articular cartilage-like bilayer hydrogel-coated ultra-high molecular weight polyethylene (BH-UPE) was built up through swelling-driven surface adsorption and UV polymerization (Figure 6b) [54]. The UPE was impregnated with a hydrophobic photoinitiator (benzophenone, BP) through swelling-driven surface absorption in dimethyl formamide (DMF) solution and then covered with a homogeneous precursor solution containing acrylamide (AM) and methyacrylate (MA) comonomers, a N,N′-methylenebisacrylamide crosslinker, and a hydrophilic photoinitiator (2-hydroxy-4′-(2-hydroxyethoxy)-2-methylpropiophenone) in deionized water. Following irradiation with ultraviolet light for polymerization, a unique bilayer poly(AM-co-MA) hydrogel coating was formed on the UPE surface. In a reciprocating sliding motion against a 6.5 mm-diameter steel ball in a ball-on-disk tribometer, the COF drops substantially (by ca. 80%) from around 0.034 for untreated UPE to less than 0.007 for BH-UPE under a load of 2 N. Huang et al. attribute this reduction in the COF to a stable hydration layer formed on the hydrophilic porous surface during contact, thus maintaining an efficient boundary lubricating layer.

In sum, FRP is commonly used in the synthesis of a wide range of polymers, including linear, random, and crosslinked polymers, frequently exploited in a lubrication context. While it presents several advantages such as simplicity, versatility, and the capability to polymerize a diverse array of monomers under various conditions, it also poses certain challenges, including difficulties in controlling the molecular weight (MW), molecular weight distribution (MWD), and designing the block copolymer structure [55].

### 3.2. Controlled/Living Radical Polymerization (CRP)

**Figure 7:**
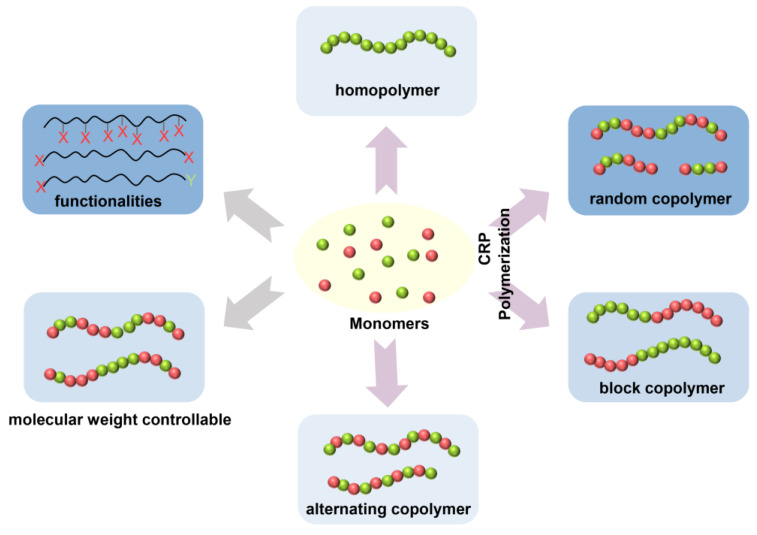
Examples of molecular structures attained through conventional free radical polymerization (CRP).

Conventional free radical polymerization (FRP) has a number of disadvantages, such as poor control of the degree of polymerization, polydispersity, end functionalities, chain architectures, and compositions. Controlled/living radical polymerization (CRP) is equipped with such control, paving the way for an exceptional opening in materials design, enabling the aptitude to construct bioconjugates, inorganic–organic hybrid materials, and surface-anchored copolymers (Figure 7) [56]. The three main CRP methods are as follows: stable free radical polymerization (SFRP), transition metal-catalyzed atom transfer radical polymerization (ATRP), and reversible addition fragmentation chain transfer (RAFT) polymerization.

#### 3.2.1. Atom Transfer Radical Polymerization (ATRP)

**Figure 8:**
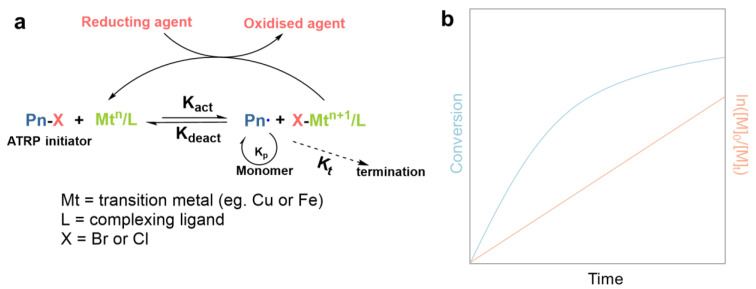
(**a**) Transition metal-catalyzed ATRP. (**b**) Visual illustration of the relationship of the conversion vs. time in linear and semilog plot [58]. (**a**,**b**) Reprinted (adapted) with permission from Ref. [58]; Copyright © 2001 American Chemical Society.

Atom transfer radical polymerization (ATRP), a living polymerization technique, is a versatile synthetic tool in designing well-defined polymeric materials. The development of ATRP involved the exploitation of an applicable catalyst (transition metal compound and ligands), selection of an initiator with the appropriate framework, and optimization of the polymerization conditions, resulting in a linear increase in molecular weights as conversion progressed and the narrow polydispersity, which were typical of a living process [57]. A universal mechanism for ATRP is described in Figure 8a. The radicals, or the active species, are generated from the activation of initiator molecules, often alkyl halide (R-X), through a reversible redox process catalyzed by a transition metal complex (Mt^n^/ligand). Upon the addition of intermediate radicals to monomer molecules (typically vinyl monomers), new carbon–carbon bonds formed and the polymer chains propagated while the active radical species simultaneously regenerated. This allowed for precise control over the polymer architectures (such as linear, star, comb, branched, grafted, block, gradient, alternating), and the ability to incorporate various functional groups into the polymer chains. The conventional linear variety of conversion with time in a semilog plot (Figure 8b) indicates that the concentration of active species remains constant and has the characteristics of first-order kinetics relative to the monomer during polymerization [58].

**Figure 9:**
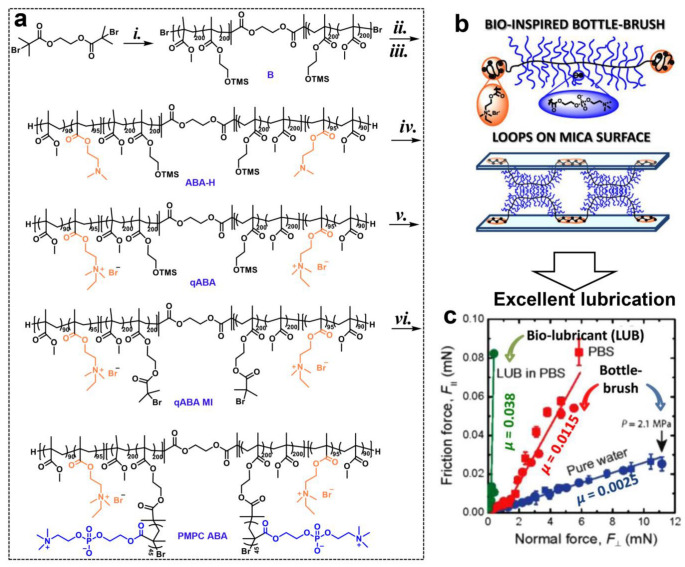
(**a**) Synthesis of lubricin (LUB) mimicking ABA polymer: (*i*) MMA, HEMA-TMS, CuBr/CuBr_2_/dNbpy, anisole, 40 °C; (*ii*) MMA, DMAEMA, CuCl/CuCl_2_/dNbpy, anisole, 60 °C; (*iii*) CuBr/TPMA, SnBu_3_H, anisole, rt; (*iv*) bromoethane, acetone, 0 °C-rt; (*v*) 2,6-DTBP, KF, TBAF, BiBBr, dry THF, 0 °C–rt; (*vi*) MPC, CuCl/CuCl_2_/bpy, methanol, 30 °C. (**b**) The architecture of bottle-brush polymer mimicking LUB absorbed on the surface of mica. (**c**) The friction force vs. normal force results (Amontons-like behavior) of the mica coated with ABA polymer loops in pure water and PBS [37]. Reprinted (adapted) with permission from Ref. [37]; Copyright © 2014 American Chemical Society.

As an example of the versatility of ATRP, we describe its use to synthesize a molecule inspired by the structure of lubricin (LUB). LUB is a proteoglycan secreted in joints which plays an important role in cartilage lubrication [59]. Its structure broadly consists of two positively-charged end-domains joined by a central negatively-charged, carbohydrate-rich, bottle-brush-like central domain. The LUB mimicking the ABA bottle-brush polymer was synthesized in six steps via a combination of ATRP and post-modification techniques (Figure 9a) [37]. To mimic the LUB architecture, the synthetic polymer incorporates two primary topological features (Figure 9b): (1) the central water-soluble and biocompatible bottle-brush domain (B), leading to effective lubrication behavior, and (2) the outer linear segments (A) with positively charged moieties, serving as anchoring blocks for adsorption on a mica surface, for determining its lubrication properties in an SFB. The flexible, densely grafted B segment, composed of a flexible methacrylate backbone (with a degree of polymerization (DP) of ~800) decorated with poly(2-methacryloyloxyethyl phosphorylcholine) (PMPC, DP~45) brushes, was attained through a “grafting from” method. This segment has a fully extended chain with a length of approximately 200 nm, resembling that of LUB. The PMPC side chains provide extremely good lubrication under physiological pressure (between 2–7.5 MPa), as known from earlier studies [60], and excellent biocompatibility. The di-initiators at the end of the chain (B) underwent chain extension with copolymers of (2-dimethylamino)ethyl methacrylate (DMAEMA) and methyl methacrylate (MMA), resulting in the formation of the A segment (DP~185) in the ABA triblock copolymer. Finally, the quaternary ammonium cations of DMAEMA segments in the ABA triblock copolymer were formed by a quaternization reaction with ethyl bromide, achieving a strong adsorption on negatively charged mica surfaces. Thus, the ABA triblock polymers generated loops when they absorbed on the surface of the mica (Figure 9b), yielding a COF of about 0.0025 in pure water and 0.0115 in PBS solution as measured by SFA (Figure 9c). Importantly, this synthetic pathway provides access to a wide range of ABA bottle-brush analogs, offering excellent control over topological factors such as the domain size, composition, length, or packing density of grafts, which are crucial for designing efficient artificial lubricants.

**Figure 10:**
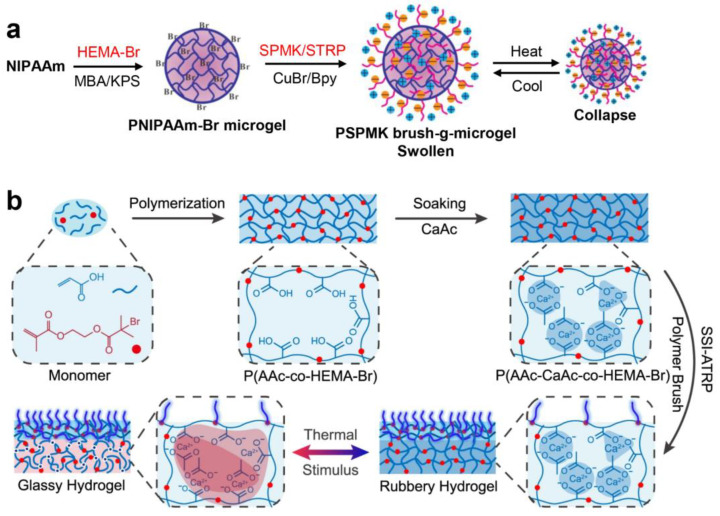
(**a**) The preparation of PSPMK brushes-grafted PNIPAAm hairy spherical microgels, and the drug loading and release of the hairy spherical microgels triggered by heat [7]. (**b**) Schematic representation of MALH preparation and the mechanism behind the thermal-triggered modulus change. The phase transition of MALH from a soft gel to a rigid state is due to the dehydration of the hydrophobic residues (acetate) in the MALH network at high temperature, inducing phase separation and enhancing the electrostatic interaction of the nearby charged residues (Ca^2+^-COO^−^) [61]. (**a**) Reprinted (adapted) with permission from Ref. [7]; Copyright © 2014 American Chemical Society. (**b**) Reprinted (adapted) with permission from Ref. [61]; Copyright © 2022, The author(s).

Polymer brushes with versatile properties can be grafted from micro- and bulk hydrogels via ATRP. Liu et al. [7] fabricated negatively charged poly(3-sulfopropyl methacrylate potassium salt) (PSPMK) brushes grafted thermosensitive poly(N-isopropylacrylamide) (PNIPAAm) microgels via ATRP, as shown in Figure 10a. The negatively charged PSPMK brushes as the outer layer of microgels are highly hydrated and fully stretch in aqueous solution, which facilitates hydration lubrication and stabilizes the colloid due to electrostatic and steric interactions. Frictional tests were conducted in a ball-on-block setup under reciprocating mode, featuring a temperature-regulated stage. Contact between the frictional pair was established by forcing the upper running ball (PDMS hemisphere with a diameter of 10 mm) against the lower stationary disk (silica wafer treated with oxygen plasma), which reciprocated at a predefined sliding velocity (100 mm/min) and normal load (1~10 N) for 100 cycles. A shear-thickening nature was observed in this hairy (i.e., brush-bearing) microgel owing to the stronger stretching of PSPMK brushes at an increased shear rate causing a looser colloidal arrangement on a microlevel. In addition, the hydrated PSPMK brushes endow the microgels with a COF of 0.005–0.015 under a range of normal loads (1–10 N), and avoid interpenetration due to the steric repulsion arising from chain configurational entropy. A form of modulus adaptive lubricating hydrogel (MALH, exhibited in Figure 10b), consisting of the top hydrophilic poly(3-sulfopropyl methacrylate potassium) (PSPMA) brushes (mimicking the slippery surface mucus of fish) and the bottom poly(AAc-CaAc-co-HEMA-Br) hydrogel (mimicking the muscle-stiffening mechanism of fish) was reported by Zhang et al. [61]. The sphere-on-disk tribometer was utilized to measure the COF of an MALH hydrogel under different conditions (20 and 80 °C). MALH hydrogel possesses a switchable lubrication behavior with a high COF (~0.37) at 20 °C and a low COF (~0.08~0.027) at 80 °C resulting from the elastic modulus change in the bottom phase-separation hydrogel from the soft state (~0.3 MPa, 20 °C) to the rigid state (~120 MPa, 80 °C), for which the deformation dissipation (F_def_) significantly reduced during friction.

ATRP is a well-known technique for the synthesis of molecules with complex and well-defined architectures, low dispersity, and precise molecular weight. However, ATRP typically demands rigorous oxygen exclusion, rendering it a time-consuming and challenging technique for nonexperts to implement [62]. Developing oxygen-tolerant ATRP, like enzyme-assisted and activator regenerated by electron transfer (ARGET) ATRP, in designing lubricating polymer materials is an emerging direction [62,63].

#### 3.2.2. Reversible Addition-Fragmentation Chain-Transfer (RAFT)

**Figure 11:**
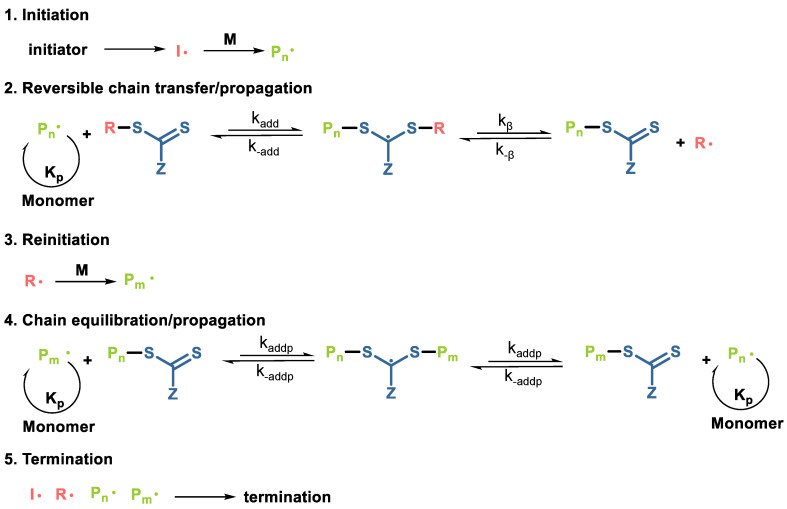
Mechanism of reversible addition–fragmentation chain transfer (RAFT) polymerization. The RAFT process begins with the activation of the initiator (Step 1), during which the initiator decomposes to form two fragments (I·). These fragments then react with monomer molecules (M) to yield a propagating polymeric radical (P_n_·). P_n_· subsequently adds to the RAFT agent (chain transfer agent, CTA) to generate a RAFT adduct radical (Step 2), which is capable of losing either the R group (R·) or the polymeric species (P_n_·). Through a process of rapid interchange, an equilibrium is established between the active propagating radical and dormant species (Step 4), allowing chains to have equal opportunities for growth and leading to a narrow PDI [65].

What distinguishes RAFT polymerization from all other CRP methods is that it can be applied to various monomers and reaction conditions, as well as endowing polymers with a well-controlled molecular weight and narrow polydispersity (usually <1.2; sometimes <1.1) [64]. The mechanism of RAFT polymerization includes a reversible addition-fragmentation sequence, where the transfer of the S=C(Z)S- segment between active and dormant chains guarantees the living character of the polymerization (Figure 11) [65]. This technique allows precise control over the polymer chain length, end-group functionality, and composition, enabling synthetic polymers with tailored architectures, such as block copolymers, star polymers, and gradient copolymers.

**Figure 12:**
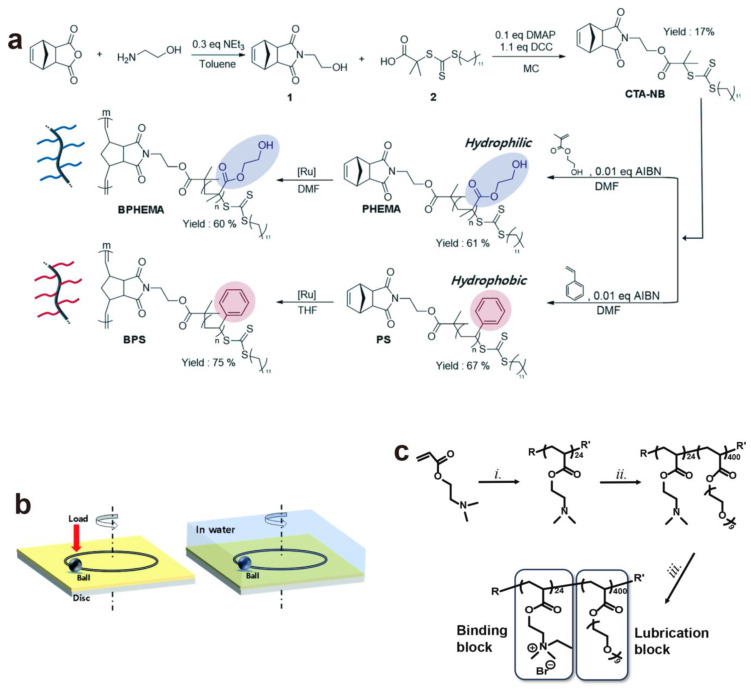
(**a**) Synthetic route of the bottle-brush polymers BPHEMA and BPS. (**b**) The illustration of ball-on-disc tests in dry and wet conditions [38]. (**c**) Synthesis of a lubricin-mimetic di-block copolymer: (*i*) 4,4′-azobis(4-cyanopentanoic acid) (ACPA), 4-cyano-4-(phenyl-carbonothioylthio)pentanoic acid (CPADB), anisole, 70 °C; (*ii*) ACPA, DMAEA, anisole, 65 °C; (*iii*) EtBr, acetone, room temperature [66]. (**a**,**b**) Reproduced from Ref. [38] with permission from the Royal Society of Chemistry. (**c**) Reproduced from Ref. [66]; Copyright © 2019 National Academy of Sciences.

The bottle-brush polymer BPHEMA and BPS were synthesized, respectively, using hydrophilic HEMA and hydrophobic styrene monomers via RAFT and ring-opening metathesis polymerization (ROMP) (Figure 12a) [38]. To analyze their hydration lubrication properties, BPHEMA and BPS coated on Si-wafers via spin-coating were mounted onto a ball-on-disk tribometer (Figure 12b). The friction coefficient was evaluated under a load of 1 N in water at room temperature, the results of which suggest that polymers with a greater hydrophilicity exhibit a stronger hydrogen bonding network and a lower COF. This is attributed to the presence of a thicker water lubricating layer on hydrophilic surfaces. A di-block copolymer consisting of a cationic cartilage-binding domain (polyacrylic acid (PAA) backbone decorated with quaternary ammonia groups, Mn ~3 kDa) and a brush-lubricating domain (polyethylene glycol (PEG), Mn ~200 kDa), which facilitates the polymer to conserve water and to resist compression, was synthesized by sequential RAFT polymerization (Figure 12c) [66]. The cartilage surface incubated in PBS and then in polymer solution (in PBS) was loaded onto a custom-built tribometer [67], whose friction was evaluated in a PBS solution under boundary mode with 30% compressive strain and a linear oscillation velocity of 0.3 mm/s. The copolymer-coated cartilage samples resulted in a decrease in the COF from ca. 0.4 (bare articular cartilage) to 0.09 (cartilage coated with polymers), a level equivalent to that of lubricin (ca. 0.09), though care must be taken when comparing absolute friction values on ex vivo cartilage whose surface properties differ from the in vivo tissue.

### 3.3. Click Chemistry

“Click chemistry”, the goal of which is to develop series of powerful, dependable, and selective reactions for the swift synthesis of functional new compounds, was first proposed by Sharpless in 2001 [68,69]. There are certain strict criteria that the reactions must fulfill to be useful: being fast, modular, wide in scope, high yielding, processable under simple reaction conditions (insensitive to oxygen and water), generating minimal by-products, and stereospecific. It includes four classes of chemical transformations: cycloadditions of unsaturated species (1,3-dipolar cycloaddition reactions and Diels–Alder cycloaddition), nucleophilic substitution chemistry (ring-opening reactions), carbonyl chemistry of the “non-aldol” type (the generation of thioureas, aromatic heterocycles, oxime ethers, hydrazones, and amides), and additions to carbon–carbon multiple bonds (Michael additions, sulfenyl halide addition, epoxidation, dihydroxylation, and aziridination). 

**Figure 13:**
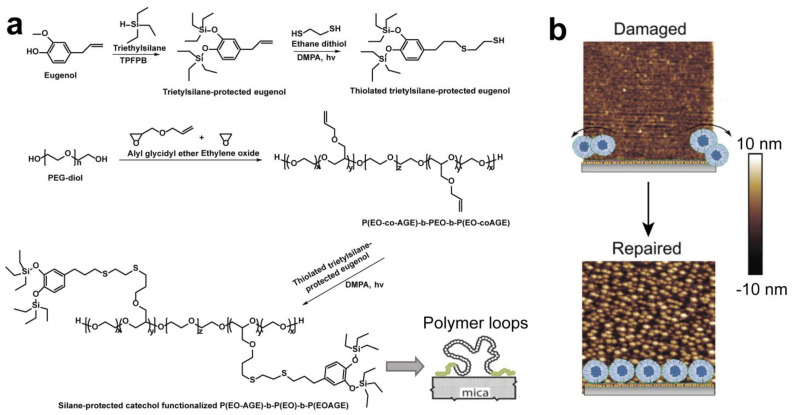
(**a**) The synthesis of a silyl-protected catecholic monomer and catechol-functionalized PEG triblock copolymer [39]. (**b**) AFM images of damaged and repaired F127-B micelle-anchored surfaces [72]. (**a**) Reprinted (adapted) with permission from Ref. [39]; Copyright © 2015 American Chemical Society. (**b**) Reprinted (adapted) with permission from Ref. [72]; Copyright © 2023 The authors. Advanced Functional Materials published by Wiley-VCH GmbH.

A thiol-ene reaction is an organic reaction between a thiol (R-SH) and an alkene (R_2_C=CR_2_) to produce a thioether (R-S-R’), defined as a click chemistry reaction on account of the characteristics of a high yield, stereoselectivity, high reaction rate, and thermodynamic driving force [70]. Kang et al. [39] prepared a catechol-functionalized triblock copolymer that has a neutral poly(ethylene oxide) (PEO) middle block and two catechol-functionalized end blocks via a sequence of ring-opening and thiol-ene reactions. As shown in Figure 13a, the synthetic process of the triblock polymer starts with the catechol-functionalized PEO-based triblock copolymer. Triethyl silane-protected eugenol was synthesized through a condensation reaction between hydrosilanes (Si-H) and alcohols (R-OH), ethers (R-OR′) catalyzed by tris(pentafluorophenyl)borane (TPFPB) at room temperature, and then the compound was functionalized with thiol via a thiol-ene reaction with ethanedithiol. Following the anionic ring-opening copolymerization of ethylene oxide (EO) and allyl glycidyl ether (AGE) from a PEO macroinitiator, P(EO-co-AGE)-b-PEO-b-P(EO-co-AGE) was synthesized in THF at 45 °C. The prepared thiolated triethylsilane-protected eugenol was then deployed to functionalize the P(EO-co-AGE)-b-PEO-b-P(EO-co-AGE) triblock copolymers via a simple thiol-ene reaction under photochemically induced radical conditions. These triblock polymer loops substantially reduce the COF between the mica surfaces to 0.002 ± 0.001 and 0.004 ± 0.001 in the pH range from 3 to 7, mainly by reducing layer interpenetration. 

The dynamic covalent bond, a key aspect of click chemistry, has emerged as a sophisticated molecular design strategy largely characterized by its reversibility and selectivity, which has attracted much attention in molecule recognition, biomolecule assembly, and intelligent materials [71]. Superlubricating surfaces with targeted self-repairability were attained by click chemistry under mild conditions [72]. Poly[(2-Methacryloyloxy ethyl trimethylammonium)-co-(2-lactobionamidoethyl methacrylamide)] (P(MTAC-co-LAEMA)) and poly[(2-Methacryloyloxy ethyl trimethylammonium)-co-(2-Aminoethylmethacrylamide)] (P(MTAC-co-AEMA)) copolymers were utilized as bridges between F127 triblock copolymer micelles (boronic acid-ended F127-B and aldehyde group-ended F127-CHO) and mica surfaces through the electrostatic attraction and click chemistry, where P(MTAC-co-LAEMA) and (P(MTAC-co-AEMA) copolymers bind on the negative mica surface via electrostatic attraction due to the positively charged quaternary ammonium groups in MTAC, and F127 micelles carry out self-healing on P(MTAC-co-LAEMA) and P(MTAC-co-AEMA) decorated mica surfaces via boronate esterification and nucleophilic addition reactions. Surface force measurements (using an SFB) were used to probe the interaction forces and lubrication behaviors between F127-B and F127-CHO micelle-anchored surfaces. A strong and long-range repulsion starts from about 50 nm corresponding approximately to the total hydration diameters of the F127-B and F127-CHO micelles. The micelles-coated mica surfaces display superlubricity (COF µ ≈ 0.002) in PBS buffer containing K^+^ ions at a physiologically high pressure of ≈7.5 MPa. This was attributed to the strong steric repulsion generated from the polymer brush-like nature of the opposing swollen micelles and the abolishment of cation-mediated adhesion. Based on the reversibility of dynamic covalent bonds, the surfaces maintained a high lubricity after damage–repair over 20 cycles. AFM imaging (Figure 13b) verified that the anchored micelles can self-repair after they were scratched off from the substrate surfaces due to dynamic covalent bonds.

## 4. Supramolecular Assembly

Supramolecular assembly refers to the spontaneous organization of molecules into complex and functional structures through weaker noncovalent interactions such as hydrogen bonding, van der Waals forces, π-π stacking, and electrostatic interactions [73]. These interactions allow molecules to self-assemble into various architectures, ranging from simple dimers to intricate nanoscale structures, examples of which include micelles, host–guest complexes, metal–organic frameworks (MOFs), supramolecular gels, and nanoparticles [4,74,75]. These assemblies are of considerable interest in various fields, including materials science, nanotechnology, drug delivery, regenerative medicine, and tissue engineering, due to their potential in creating functional materials with tailored properties [76]. Supramolecules with spacious cavities and intricate 3D networks, especially, can effectively diminish interfacial friction and wear in materials.

**Figure 14:**
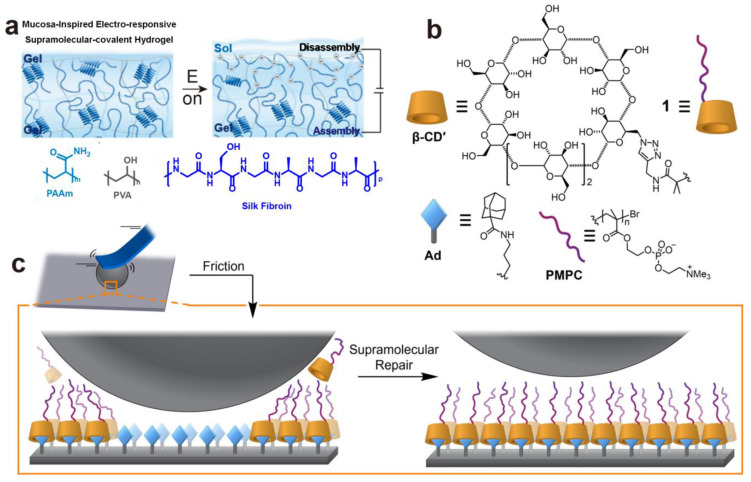
(**a**) The schematic diagram of the electro-responsive mechanism of the mucosa-inspired hydrogel under an electric field [40]. (**b**) The molecular structural formulas of the components of the hydration lubrication surface. (**c**) The self-healing mechanism of the β-CD-PMPC polymer on the surface coated with Ad units via noncovalent bonds after wear [41]. (**a**) Reprinted (adapted) with permission from Ref. [40]; Copyright © 2023 Wiley-VCH GmbH. (**b**,**c**) Reprinted (adapted) with permission from Ref. [41]; Copyright © 2021 Elsevier Inc.

Wenlong Song’s group developed a mucosa-inspired electro-responsive hydrogel consisting of an electro-responsive supramolecular noncovalent section (silk fibroin) and a covalent polymer interpenetrating network (polyacrylamide/polyvinyl alcohol double network (PAAm/PVA)) [40]. When an electric field is applied to the supramolecular hydrogel, it generates a partial gel-sol transition due to the disassembly of the silk fibroin network (Figure 14a). The sol state layer on the hydrogel surface, acting as a lubricating layer, decreases the COF to below 0.08. This group also incorporated a supramolecular thixotropic N-Fluorenylmethoxycarbonyl-L-tryptophan (FT) hydrogel into a PAAm/PVA network [77]. The gel-sol transition of thixotropic FT hydrogel activated by shear force conducts a lubricating function, while the robust double network PAAm/PVA hydrogel serves as the physical support. Under continuous shear, the COF on the FT-PAAm/PVA hydrogel surface would reduce owing to the disassembly of FT.

Taking advantage of the dynamic reversibility and specific host–guest interactions, dynamically repairing surfaces exhibiting hydration lubrication can be achieved, based on the self-assembly of polymers in water. In one host–guest complex (Figure 14b) [41], alkyne-terminated poly(2-methacryloyloxyethyl phosphorylcholine) (PMPC)-decorated mono-(6-azido-6-deoxy)-b-cyclodextrin (β-CD’) via Cu(I)-catalyzed azide-alkyne cycloaddition (CuAAC) serves as a lubricating host, while a 1-adamantane carboxamide (Ad) groups-functionalized Ti-6Al-4V (Ti) wafer surface acts as a guest. It shows that surfaces undergo self-healing following mechanical wear via noncovalent bonding interactions, which, in turn, restores lubricity rapidly (Figure 14c). Eventually, a low-COF (µ = 0.024–0.028) hydration lubrication layer that is capable of spontaneously dynamic repair was obtained by these supramolecular host–guest interactions. 

In sum, supramolecular assembly with its versatility in creating complex and functional structures from simple building blocks allows for the design and fabrication of materials with tailored properties for specific applications under mild conditions, enabling the formation of structures without the need for harsh chemical reactions or high temperatures. Furthermore, supramolecular assemblies can be dynamic and responsive to external stimuli, offering opportunities for reversible changes in structure and properties; this is beneficial for applications such as drug delivery, sensing, and self-healing materials. However, this approach, while promising, suffers from inherent limitations (unsatisfactory mechanical performance and poor durability) that may hinder its applicability [78].

## 5. Lubrication Mechanism of Polymer-Based Lubricants

Polymer-based lubricants may act in two complementary mechanisms: entropic factors arising from excluded volume interactions limit the interpenetration of brush-like chains and thus reduce frictional dissipation [60]. At the same time, hydration lubrication, as postulated by Klein [25], which relies on the rapid relaxation dynamics of the hydration water molecules tenaciously held by charged monomers in spite of the applied stress and the restricted geometry, acts at a slip plane between hydrated polymer chains where their monomers are in contact. 

## 6. Conclusions

Synthetic polymer-based lubricating gels/coatings are often more favorable for large-scale applications due to their lower cost, relative ease of production, and facile tunability of structures and functions. This review systematically introduced some of the main synthetic strategies, and the corresponding measurements of the resulting reduced-friction materials. Nonetheless, while progress has been made in the field of functional lubricating polymer gels/coatings, there are still several challenges to be addressed.

Firstly, there are still questions regarding the overall lubrication mechanism of polymer gels/coatings. Although the hydration lubrication mechanism—whereby angstrom-thick hydration layers form fluid yet tenaciously held lubrication elements [24]—provides an organizing principle at the molecular level, it is well recognized that the overall lubrication by polymer gels/coatings is determined across many length scales [79] (and not just at the sub-nanometer level of the hydration layers). Despite the ongoing utilization of SFB and AFM nanotribological measurement technologies, as well as emerging theoretical simulation methods in friction research [80], all of which provide molecular-level information, a primary materials emphasis concerns specific-design systems such as polymer brushes and lipid assemblies (liposomes or bilayers). It is still difficult to uncover the effect of the architectures and deformation of complex polymeric systems on the reduction in frictional dissipation and understand the lubrication mechanisms at a macromolecular or higher level. Thus, real-time observations of dissipation processes and an intensive comprehension of the stimulation and feedback behaviors of the polymer-based lubricants during friction are still required. Lin et al. reviewed the prospect of advanced friction measurements, such as the combination of infrared spectroscopy and SFB, which will improve the design of lubricating polymer gels/coatings at macromolecular scales [29].

Secondly, although various types of lubricating polymer assemblies have been synthesized using free radical polymerization (FRP), controlled/living radical polymerization (CRP), click chemistry, and supramolecular chemistry, important concerns need to be addressed. These include the need for complicated chemical procedures, poor wear resistance, and biodegradability of polymeric lubricants. Recently, there is growing concern about plastic pollution and global warming; thus, developing lubricating polymer gels/coatings with specific chemical structures that minimize their wear on the one hand (through improved lubricity, for example), and facilitate their recycling on the other, is crucial for addressing the worldwide plastic waste predicament. For example, polymers with easily cleavable bonds or those that can be depolymerized into monomers under mild conditions are preferred [81,82]. By advancing recyclable polymer design and recycling processes, we can move towards a more sustainable approach to plastic usage and waste management. Moreover, considering the paramount commercialization, the large-scale and efficient synthesis methods of lubricating polymer gels/coatings need to be explored systematically. Reported polymer gels/coatings are often sophisticated chemicals suitable only in the laboratory, demanding intricate multi-step synthesis, separation, and purification processes. Therefore, it is necessary to try to simplify the materials’ synthesis route, while maintaining the high lubrication performance of future polymer gels/coatings. 

Last but not least, despite extensive progress in lubricating polymer gels/coatings, only a limited number of cases have systematically examined the relation of surface topology to enhanced lubrication performance. Inspired by nature, particularly its solutions in highly stressed environments such as the articular cartilage in hip or knee joints, manipulating the surface topology of materials has emerged as a viable approach to regulate their lubrication [48,51]. Some sophisticated preparation technologies (i.e., 3D/4D printing and template methods) present significant opportunities to manipulate lubricating polymer gels/coatings with multiscale surface topologies to establish the relation of these to their lubricity, or to create polymeric tissue engineering scaffolds with precisely positioned lubricating surfaces. Such developments may greatly increase the scope of future lubricating polymer applications.
